# Draft Genome Sequence of Bacillus subtilis SB-14, an Antimicrobially Active Isolate from Namibian Social Spiders (*Stegodyphus dumicola*)

**DOI:** 10.1128/MRA.00156-19

**Published:** 2019-06-20

**Authors:** Stine Sofie Frank Nielsen, Simone Weiss, Seven Nazipi, Ian P. G. Marshall, Trine Bilde, Andreas Schramm

**Affiliations:** a Department of Clinical Medicine, Aarhus University, Aarhus, Denmark; b Department of Bioscience, Section for Microbiology, Aarhus University, Aarhus, Denmark; c Department of Bioscience, Section for Genetics, Ecology and Evolution, Aarhus University, Aarhus, Denmark; Broad Institute

## Abstract

We present the high-quality draft genome sequence of Bacillus subtilis SB-14, isolated from the Namibian social spider Stegodyphus dumicola. In accordance with its antimicrobial activity, both known and potentially novel antimicrobial biosynthetic gene clusters were identified in the genome of SB-14.

## ANNOUNCEMENT

Increasing antibiotic consumption has accelerated resistance development, prompting the need for novel antibiotics (1, 2). Stegodyphus dumicola spiders are social spiders that live in communal nests with a high degree of inbreeding and low genetic variation, making them potentially vulnerable for pathogen attack. We therefore suggest an intricate symbiotic relationship with a well-developed protective microbiome. This microbiome may present an unexplored source of antibiotics (3, 4).

Bacillus subtilis SB-14 was isolated from the body surface of a *Stegodyphus dumicola* spider, as follows: a spider was immersed in nutrient broth (Sigma-Aldrich), which was incubated at 30°C overnight, plated onto nutrient broth agar plates, and grown aerobically at 30°C. Single colonies from serial dilutions were restreaked until a pure culture was established. Colonies were screened by colony PCR and 16S rRNA gene sequencing (5). The antimicrobial activity of SB-14 against Staphylococcus epidermidis BMC-HMP0060 was detected using the Kirby-Bauer disk diffusion susceptibility test protocol (6), with the modification that SB-14 was point inoculated onto a lawn of S. epidermidis.

Genomic DNA was extracted using the DNeasy blood and tissue kit (Qiagen) and prepared for sequencing with a Nextera XT DNA library prep kit (Illumina). Sequencing was performed utilizing an Illumina MiSeq platform with a paired-end 300-bp read MiSeq reagent kit, yielding ∼5.5 million sequencing reads in total (∼1.11 Gbp), with approximately 260× coverage. Read quality was analyzed by FastQC version 0.11.7 (https://www.bioinformatics.babraham.ac.uk/projects/fastqc/), and reads were trimmed by Trimmomatic version 0.36 (7) using length-based trimming with the following parameters: headcrop, 20; crop, 290; a minimum average quality score of 20; and a 4-bp sliding window. Finally, the genome was assembled using SPAdes version 3.11.1 (8), resulting in 37 scaffolds, of which 31 were ≥200 bp when applying the parameters –careful -k 21, 33, 55, 77, 99, 127.

CheckM version 1.0.9 (9) estimated 98.28% completeness and 0.00% contamination of the SB-14 genome, using the gene marker set for the domain *Bacteria*; the corresponding values using the gene marker set for Bacillus subtilis were 97.29% and 2.63%, respectively.

NCBI BLAST (10) analysis of the 16S rRNA gene revealed 99% identity to the 16S rRNA genes of multiple Bacillus subtilis strains. This genus and species identity was confirmed by an average amino acid identity of >98% (11), average nucleotide identity of >98% (11), and 94.18% digital DNA-DNA hybridization value (http://ggdc.dsmz.de/), compared to Bacillus subtilis subsp. inaquosorum (NCBI RefSeq accession no. NZ_CP013984).

The draft genome of Bacillus subtilis SB-14 contains 4,262,181 bp on 31 scaffolds of ≥200 bp, with an average G+C content of 44.02% and an *N*_50_ value of 781,251 bp. Prokka version 1.12 (12) analysis found 81 tRNA, 10 rRNA, and 4,210 protein-coding sequences. Upon antiSMASH version 3.0 (13) analysis, 30 secondary metabolite biosynthetic gene clusters were identified. Five of these were 100% identical to known clusters encoding the biosynthesis of teichuronic acid (14), bacillibactin (15), the antifungal lipopeptide fengycin (16), and the antibiotics bacillaene (17) and subtilosin A (18). Three other clusters had 82%, 40%, and 18% identity to clusters known for the biosynthesis of the antimicrobial metabolites surfactin (19), bacillomycin (20), and zwittermicin A (21), respectively; these three may therefore represent novel variations of these metabolites.

### Data availability.

The isolate Bacillus subtilis SB-14 has been deposited at the DSMZ (https://www.dsmz.de/) under accession no. DSM 109343. This whole-genome shotgun project has been deposited in DDBJ/ENA/GenBank under BioProject accession no. PRJNA438195. The raw data can be found under accession no. SRR8560781, and the assembled genome has GenBank accession no. PXUR00000000. The version described in this paper is the first version, PXUR01000000.
